# New Method Improves the Assessment of Aortic Regurgitation Grade during TAVR by Aortography

**DOI:** 10.5935/abc.20180158

**Published:** 2018-08

**Authors:** Henrique B. Ribeiro

**Affiliations:** Instituto do Coração de São Paulo - Universidade de São Paulo, São Paulo, SP - Brazil

**Keywords:** Transcatheter Aortic Valve Replacement/methods, Aortic Valve Insufficiency/mortality, Aortic Valve Insufficiency/diagnostic imaging, Echocardiography, Three-Dimensional/methods, Aortography/methods

Transcatheter aortic valve replacement (TAVR) is a rapidly expanding alternative to surgical aortic valve replacement for patients deemed inoperable or at high or intermediate operative risk. Yet, residual aortic regurgitation (AR) secondary to paravalvular leaks (PVL) remains a procedural limitation.^1^ Although residual AR after TAVR is frequent, affecting up to approximately 70% of the treated patients,^2^^-^^4^ it is moderate to severe in approximately 12% of these^4^ and steadily below 5% with current-generation devices, which come with specific sealing features.^1^ Noteworthy, moderate/severe AR has a detrimental clinical impact after TAVR, with a 3-fold increase in 30-day mortality and a 2.3-fold increase in 1-year mortality.^4^ Thus, its accurate assessment and quantification with a multimodality approach is key for appropriate utilization of additional procedures to reduce PVL, such as balloon post-dilatation (BPD), valve-in-valve, or leak closure.^1^^,^^5^

While Doppler echocardiography has been the most common method to assess AR following TAVR, its accurate quantification is challenging since AR jets are often multiple and eccentric.^3^^,^^5^^-^^7^ Therefore, other methods for proper AR assessment have been evaluated in recent years, such as 3D echocardiography, hemodynamic AR index, aortography and even cardiovascular magnetic resonance, each one with its specific advantages and disadvantages.^1^^,^^5^^,^^7^

In the current issue of the journal, Miyazaki et al.^8^ investigate a quantitative angiographic assessment of AR by videodensitometry (VD-AR) before and after BPD was performed. VD-AR was shown to decrease significantly from 24.0 [18.0-30.5] % to 12.0 [5.5-19.0] % (p < 0.001) after BPD, with some degree of AR grade improvement for up to 70% of patients treated. Of note, significant AR (VD-AR > 17%) was observed in 47 patients (77%) before and in 19 patients (31%) after BPD; moreover, in up to a quarter of these, pre-BPD VD-AR was below 17%, indicating that this additional maneuver could have been avoided. The study has its inherent limitations, e.g., the cohort was relatively small, with retrospective patient selection and imaging acquisition, and the decision whether or not to perform BPD was left to the discretion of the operators. Accordingly, the study only comprises cases where BPD was deemed necessary, and only aortograms with good quality imaging were selected.

Notably, the technique used to quantify VD-AR is a novel method that can accurately determine the regurgitation fraction in aortograms performed during TAVR; it uses dedicated software and showed excellent reproducibility and accuracy.^9^^,^^10^ This technique provides an accurate assessment of the severity of PVL, and a VD-AR index greater than 17% correlated with increased mortality and with impaired cardiac reverse remodeling after TAVR.^11^^,^^12^ And while VD-AR measurements are performed offline only, real-time online assessment is underway so as to enable this method to help guiding TAVR in the near future. After all, BPD is currently performed in about 10% to 20% of patients following TAVR, and it reduces the severity of PVL by at least one grade in more than two thirds of patients.^13^^,^^14^ Nevertheless, BPD may be associated with an increased risk of cerebrovascular events and annular trauma, therefore judicious utilization of this procedure is recommended.^13^^,^^14^

In conclusion, since PVL has a negative impact on clinical outcomes after TAVR, its proper assessment through a multimodality, multiparametric, integrative approach is fundamental. Priority should be given to PVL prevention through accurate sizing of aortic annulus by 3D imaging techniques, THV devices with improved sealing features, and optimal THV sizing and positioning. Still, if PVL does occur after TAVR, the interventional cardiologist can consider corrective procedures such as BPD, valve-in-valve, or leak closure. The novel VD-AR after TAVR also allows quantitatively assessing post-TAVR regurgitation and may assist decision making on whether or not to perform BPD, as well as determining its efficacy. Future prospective studies are warranted to further confirm the present results.
